# Tinnitus and hearing loss in people with dementia

**DOI:** 10.1002/alz.095725

**Published:** 2025-01-09

**Authors:** Mariane Gomes Machado, Thais Helena Machado, Paulo Caramelli, Luciana Macedo de Resende

**Affiliations:** ^1^ Universidade Federal de Minas Gerais, Belo Horizonte, Minas Gerais Brazil

## Abstract

**Background:**

Tinnitus has variable prevalence in the general population and is often associated with some degree of hearing loss. There are few studies on tinnitus in people with dementia. The objective of the study was to investigate the occurrence of tinnitus complaints in patients with dementia and evaluate possible relationships with hearing acuity.

**Method:**

Observational study carried out with individuals treated at a cognitive neurology outpatient clinic. The inclusion criteria were patient being monitored for dementia, patient’s ability to answer the question “Do you feel tinnitus/noise in your ears or head?” and respond to audiometry. Descriptive analysis was performed. Research Ethics Committee 5626511.

**Result:**

Overall 187 patients with mild to moderate dementia who answered the anamnesis questions. Among these patients, 70 (37.43%) reported tinnitus and had a mean age of 66.71 years, 33 (47.14%) women and 37 (52.85%) men. It was possible to perform audiometry on 62 patients, due to difficulties in carrying it out due to dementia. Of the 62 patients who complained of tinnitus and underwent audiometric evaluation, hearing loss was detected in 54 (87.10%).

**Conclusion:**

In our study, 37.43% of patients with dementia complained of tinnitus. The prevalence of tinnitus complaints in the general population varies greatly, ranging between 4.4 and 15.1% in adults and 22.2% in older adults. Changes and severity of mental status and type of dementia may worsen the perception of tinnitus. The vast majority of tinnitus patients had some degree of hearing loss. The complaint of tinnitus is strongly associated with hearing loss in previous studies. Hearing rehabilitation may be a recommended treatment for patients with dementia, hearing loss and tinnitus. In addition to improving hearing acuity, rehabilitation can reduce the perception of tinnitus, as this symptom can lead to changes in mood and behavior that are difficult to manage. The perception of tinnitus in people with dementia may be greater compared to the general population. More studies need to be carried out to better characterize the tinnitus reported by people with dementia of different types and stages to better define management by professionals.

